# Computerised Attention Functions Training Versus Computerised Executive Functions Training for Children with Attention Deficit/Hyperactivity Disorder: A Randomised Controlled Trial

**DOI:** 10.3390/jcm13237239

**Published:** 2024-11-28

**Authors:** Inbar Lucia Trinczer, Lilach Shalev

**Affiliations:** 1 Attention Lab, School of Education, Tel-Aviv University, Tel Aviv 67017, Israel; 2 Sagol School of Neuroscience, Tel-Aviv University, Tel Aviv 67017, Israel

**Keywords:** ADHD, cognitive training, small group, group-based intervention, attention, executive functions, children, active and passive control, follow-up

## Abstract

**Background**: Attention deficit/hyperactivity disorder (ADHD) is a prevalent neurodevelopmental disorder characterised by deficits in attention, hyperactivity, and impulsivity. Current treatments, such as stimulant medication and behavioural therapy, ameliorate symptoms but do not address the core cognitive dysfunctions. This study aimed to investigate the effects of two computerised neurocognitive training programs, attention functions training and executive functions training, in children with ADHD. **Methods**: Eighty children with ADHD (ages 8–13) were randomly assigned to one of three groups: Attention functions training (AFT), targeting sustained, selective-spatial, orienting, and executive attention; executive functions training (EFT), focusing on working memory, cognitive flexibility, and problem solving; or a passive control group. Training sessions were administered in small groups twice a week for nine weeks. Participants underwent comprehensive assessments of attention (Continuous Performance Test, Conjunctive Visual Search Task), executive functions (Corsi Block-Tapping Tasks), nonverbal reasoning (Raven’s Colored Progressive Matrices), parent-rated behavioural symptoms, and arithmetic performance at baseline, post-intervention, and follow-up. **Results**: The AFT group demonstrated significant improvements in sustained and selective-spatial attention, nonverbal reasoning, inattentive symptoms, and arithmetic performance, and most improvements persisted at follow-up. The EFT group showed gains in nonverbal reasoning and inattentive symptoms, although no improvements were documented in working memory or in parent ratings of executive functions. **Conclusions**: The AFT program that addressed core attentional functions in children with ADHD produced robust cognitive and behavioural benefits, whereas the EFT program yielded behavioural benefits and a limited improvement in executive functions. Future research should explore different training protocols for broader gains in executive functions. These findings support the potential of theory-driven, structured neurocognitive training targeting basic cognitive functions as an effective small-group intervention for ADHD.

## 1. Introduction

### 1.1. Background

Attention deficit/hyperactivity disorder (ADHD) is a common neurodevelopmental disorder that often causes substantial deficits in early childhood and usually persists throughout life [1]. The Diagnostic and Statistical Manual of Mental Disorders (DSM-5-TR) [2] categorises ADHD as a pattern of behaviour that occurs in a variety of settings (e.g., school, home, with friends) and can interfere with social, academic, and occupational performance. Hence, many researchers investigate a variety of treatment options for individuals with ADHD, including pharmacological approaches, behavioural therapy, psychotherapies, and biofeedback, to name a few. To date, only stimulant medications, behavioural therapy, and the combination of the two meet the criteria for empirically based treatments [3]. The scientific literature provides evidence for their therapeutic benefits, and both treatments have been shown to improve functioning to some degree and attenuate behavioural symptoms associated with ADHD [4,5,6,7,8]. However, the use of pharmacological treatment is associated with many disadvantages, such as negative parental attitudes toward these medications, physical and emotional side effects, and lack of evidence of long-term effectiveness [9,10,11]. As for behavioural therapy, it is difficult to adhere to for an extended period of time, it is expensive, and it appears that its effects do not carry over to settings or behaviours that have not been treated [5,12,13]. These drawbacks of existing treatments underscore the need for new and/or complementary treatments.

One of the available treatment options is neurocognitive training, which focuses primarily on cognitive impairments and aims to reduce the long-term negative consequences of ADHD. According to contemporary theories, neurocognitive training addresses different cognitive and attentional components as separate abilities that can be enhanced through targeted and direct training [1,14,15,16,17,18,19]. Intensive, individually adjusted structured practice of a given cognitive function (e.g., attention or memory) should lead to improvement in the trained function, which, in turn, is expected to trigger transfer and generalisation effects [14,16,20]. One of the currently available neurocognitive treatments is computer-based training. This type of training follows three important guidelines that promote continuous learning and are facilitated by the use of a computer: training tasks should begin with a low level of difficulty and gradually increase according to the individual’s performance; the training sessions should be comprised of extensive practice with repetitions; and performance should be monitored through immediate, accurate feedback [1,16,20,21]. To date, some neurocognitive training studies have reported improvement in cognitive abilities that were directly trained [15,22,23,24,25,26,27], as well as improvement in cognitive abilities that were not trained directly [14,15,23,25,27,28,29,30]. In addition, there is evidence that neurocognitive training can lead to reductions in behavioural symptoms of ADHD according to parents and/or teachers [16,18,23,27,31]. Some studies have also reported improvements in different academic outcomes [16,32], while others have not [33,34,35]. Several meta-analyses have been conducted in recent years to test whether cognitive computer training in ADHD is useful. The results of these meta-analyses [36,37,38], while documenting weak effect sizes, offer important criticisms regarding the various research methods used to date. Among the various considerations addressed, critical issues pertaining to the design of active control groups were underscored. Firstly, some studies encountered challenges in effectively concealing group affiliation. Secondly, in some studies, the employment of basic, repetitive activities throughout the intervention without variation led to a decline in the motivation of control group participants. Furthermore, several studies lacked long-term follow-up assessments, leaving inquiries about enduring effects unanswered. The present study’s design addresses these pertinent criticisms to fortify the research framework.

### 1.2. The Current Study

Many previous studies have provided evidence for the heterogeneity of the cognitive profiles of individuals with ADHD, emphasising the diverse neuropsychological profiles associated with the disorder [39,40,41,42,43,44,45,46]. Thus, from a neurocognitive perspective, the best approach to portray ADHD is to implement multidimensional models that consider various possible aetiologies that may lead to similar behavioural manifestations (see, for example, [42,44,47,48,49]).

This study is based on Tsal and colleagues’ [42] multifaceted model of attention, which refers to four distinct attention functions: (a) sustained attention, the ability to allocate attentional resources and maintain consistent performance over time, especially in simple, monotonous tasks; (b) selective-spatial attention, the ability to focus attention on relevant spatial information while suppressing adjacent distracting stimuli; (c) orienting of attention, the ability to focus attention on a specific location in the visual field, disengage, and effectively direct attention to a new location; and (d) executive attention, the ability to inhibit a prepotent response and effectively resolve conflicts between responses while ignoring salient, potentially distracting information [42,50,51]. These attention functions serve, at least to some degree, as separate cognitive functions. According to this model, a deficit in any of these functions, or a combination of deficits in different attention functions, may underlie ADHD. Accordingly, different attention profiles have been found in individuals with ADHD [42,45,52].

In addition to attention, a major line of theoretical ADHD models asserts that deficits in executive functions (EFs) serve as a major causal deficit of the disorder [47,53,54]. EFs are a set of ‘high’ cognitive abilities, including inhibition, working memory (WM), and cognitive flexibility [55,56,57]. A broader approach to EFs also incorporates planning, problem solving, reasoning, prioritising and sequencing behaviour, ignoring irrelevant information, categorising, and coping with new information or situations [58,59]. According to both approaches, EFs guide driven behaviour [56,60,61]. Studies examining the performance of EFs in ADHD have yielded mixed results, with some researchers finding EF deficits in ADHD [62,63,64,65,66,67], while others have not [68,69,70]. The current study addresses three of the above EFs: (a) cognitive flexibility, the ability to adapt quickly to changing demands or situations, to examine something from multiple perspectives, to create alternatives, and to think outside of the box; (b) working memory, a limited capacity system that allows information to be stored temporarily during a cognitive task, to retrieve information from long-term memory, to link it to new information, and to manipulate it; and (c) planning, the ability to organise behaviour in time and space in situations where the goal can only be achieved through a sequence of actions that do not individually lead to the goal [60,71,72,73,74].

The present study examined the effectiveness of cognitive training interventions for children with ADHD through two primary research questions. (1) Do attention functions training (AFT) and executive functions training (EFT) protocols produce differential improvements in cognitive performance, ADHD symptomatology, and academic achievement? (2) Are training-induced improvements maintained at three-month follow-up? These questions address key uncertainties regarding the specificity/generality and sustainability of cognitive training effects in ADHD interventions.

As mentioned earlier, one of the main criticisms of cognitive training studies conducted to date relates to the nature of activities in the active control group training sessions. In non-pharmacological intervention studies, it is challenging to produce a placebo-like treatment, i.e., a treatment that the participants perceive as a real treatment but essentially is not. For instance, when participants in the control group engage in a more monotonous activity compared to the activity in the experimental group, motivation decreases compared to participants in the research group, so differences between groups may result from mere differences in engagement and motivation rather than the unique content of the treatment. To overcome such substantial methodological limitations in previous neurocognitive training studies, the current study compared two training programs: an attention functions training (AFT) program and an executive functions training (EFT) program with a shared structure. This allowed us to maintain the anonymity of group affiliation, overcome differences in engagement and/or motivation, and distinguish between the general effect of an intervention process and the specific effects that each protocol entailed. We also included a passive control (PC) group that enabled us to rule out spontaneous developmental effects, as well as learning and familiarity effects that may result from repeated exposure to the assessment tasks.

The AFT protocol consists of the Cogmission and Computerized Progressive Attention Training (CPAT) software programs [15,16,75,76], which were developed based on leading theories and well-known methods in the field of visual attention. To address one of the main criticisms of previous cognitive training studies (i.e., simple, non-progressive training protocols for the active control group), our goal was to develop two parallel intervention protocols that promote engagement and motivation of participants in both the experimental and the active control groups. Therefore, we developed an EFT protocol that implements the same criteria that guided the design of the AFT protocol, namely, a gradual increase in difficulty levels, immediate feedback on performance, and a clear display of results that allows the trainer to facilitate training and help the participant set goals for next time. The use of two similar training protocols with a shared structure but different cognitive activities that have the potential to improve various aspects of cognitive functioning in children with ADHD is one of the strengths of the present research design. However, it also presents a major challenge in finding differential effects of the two training protocols.

Another unique characteristic of our training protocols relates to the fact that the training sessions in both groups were delivered in small groups, which added a social aspect to the training. Accordingly, the training protocols included a novel ‘small-group work’ module, based on the benefits of group therapy, that was specifically designed for them and served as a platform for facilitating meaningful social interactions. Group therapy has many benefits: it serves as a platform for peer learning through the process of interacting with other members and observing and imitating other group members; it leads to a sense of solidarity with others experiencing similar difficulties; and it fulfils the basic need for a sense of belonging by providing a safe and supportive environment to improve one’s social skills and share one’s personal story [77]. Accordingly, group therapy has been shown to have a positive impact on various outcomes, including psychosocial functioning, self-efficacy, and quality of life [78].

The group work module was carefully structured to promote social interaction and group cohesion through developmentally appropriate activities. Each session included the following. (1) An opening activity focused on getting acquainted and building trust. For example, in ‘The Wind Blows’ game, participants shared common attributes and experiences, while in ‘Our Domino’ they discovered shared interests by creating a chain of matching hobbies. (2) Two intermediate breaks featuring cooperative challenges. For instance, in the ‘Balance Keeping’ game, participants had to work together to balance a marble on an upturned cup placed on a bandana held by the entire group, and in ‘Threading the Pen’ they coordinated their movements to guide a suspended pen into a bottle using only attached strings. (3) Closing reflections focused on processing the session’s experiences as a group. Activities progressed systematically from initial trust-building exercises to increasingly complex cooperative challenges aligned with group developmental stages. Our group work module provided the framework for the training sessions of both protocols, aiming to strengthen attainment and engagement in the intervention program.

## 2. Materials and Methods

### 2.1. Participants

The sample consisted of 80 third- through sixth-grade students (mean age = 10.46, *SD* = 1.10) from local public elementary schools. The key inclusion criteria were the following: (1) ages 8–12 years old, corresponding to third through sixth grade; (2) diagnosis of ADHD according to DSM5 criteria by a qualified clinician prior to the study; and (3) provided a signed medical document confirming the ADHD diagnosis. Of this sample, 51 were boys (mean age = 10.75, *SD* = 0.97) and 29 were girls (mean age = 9.95, *SD* = 1.15). Forty-four participants were treated with stimulant medications for ADHD during the study, while thirty-six were not. Table 1 presents the distribution of participants across groups by age, gender, and medication status.

Exclusion criteria included the following: concurrent diagnosis of another mental illness or neurological disorder (other than ADHD or learning disabilities); previous severe head injury; uncorrected vision; additional developmental, sensory, or motor problems; and participation in other non-pharmacological treatment interventions specifically for ADHD (e.g., neurofeedback, cognitive–behavioural therapy, etc.).

### 2.2. Assessment Tools

The study employed multiple assessment tools to evaluate cognitive functions, behavioural symptoms, and academic performance. Table 2 provides a comprehensive overview of these measures, which are detailed below.

#### 2.2.1. Continuous Performance Test (CPT)—Sustained Attention

The CPT was used to assess sustained attention, which is the ability to maintain consistent performance over time, especially during repetitive or monotonous tasks. In this task, a series of geometric shapes of different colours were displayed on the monitor, and the participants were asked to respond to the target stimulus by pressing the spacebar and to delay their responses when other stimuli were displayed. The task consisted of a single block in which the target stimulus appeared in 70% of trials. Two measures were used to assess performance: (a) the standard deviation of response times (SD of RT) for correct responses, which reflects inconsistency in RTs; and (b) the percentage of omission errors. Higher values in these measures reflect lower sustained attention [51,79].

#### 2.2.2. Conjunctive Visual Search Task (CVST)—Selective-Spatial Attention

This task was designed to assess selective-spatial attention, reflecting one’s ability to focus on a specific spatial location while suppressing surrounding distractors [80]. In this task, participants were asked to search for a blue square that appeared among an equal number of red squares and blue circles. There were four display sizes of 4, 8, 16, or 32 items. Response times and accuracy rates were recorded for each display size with and without a target [51].

#### 2.2.3. Corsi Block-Tapping Task Forward—Visuospatial Working Memory Capacity

The computerised version we used is based on a studied visuospatial task described in the research literature [81,82] designed to evaluate visuospatial working memory capacity, which enables temporary storage and manipulation of visual–spatial information. The participants were presented with a screen of nine blocks in this task. The blocks light up in a pre-fixed sequence, and the participants were instructed to click the blocks on the screen in the same order they were lit. We calculated the summary score for this task (i.e., the longest sequence remembered multiplied by the number of correct sequences).

#### 2.2.4. Corsi Block-Tapping Task Backward—Visuospatial Working Memory Capacity

This task was also used to evaluate visuospatial working memory capacity, and the computerised version we administered implements the task described by Kessels and colleagues [83]. The task is very similar to the Corsi Block-Tapping Task Forward, except for one major difference: participants are asked to click on the blocks in the reversed order of their original presentation.

#### 2.2.5. Raven’s Colored Progressive Matrices (CPM) [84]—Nonverbal Abstract Reasoning

This task was used to assess nonverbal abstract reasoning through pattern recognition and problem solving with visual stimuli. This test is designed for children aged 5–11 years old and consists of 36 items in three sets (A, AB, B), with 12 items per set. This measure produces a single raw score that can be converted into a percentile based on normative data.

#### 2.2.6. Arithmetic Evaluation—Basic Mathematical Operations

The arithmetic task was explicitly designed for this study’s specific requirements. It consisted of 40 exercises requiring the use of the four basic arithmetic operations. In total, 20 exercises involved addition and subtraction of numerals up to 20, while the remaining 20 exercises involved multiplication and division up to 100, intermixed throughout. Participants were instructed to solve all exercises as quickly and accurately as possible. Two key measures were derived: (a) speed, quantified by the total time taken to complete the task (in seconds); and (b) accuracy, assessed by the percentage of correct answers out of 40.

#### 2.2.7. Child Behaviour Check List (CBCL)—Parent-Rated Behaviour

The parental questionnaire from the Achenbach System of Empirically Based Assessment (ASEBA) [85] assesses adaptive and maladaptive behaviours and includes eight syndrome sub-scales: (a) anxious/depressed; (b) withdrawn/depressed; (c) somatic complaints; (d) social problems; (e) thought problems; (f) attention problems; (g) rule-breaking behaviour; and (h) aggressive behaviour.

#### 2.2.8. Behaviour Rating Inventory of Executive Functioning (BRIEF)—Parent-Rated Executive Function

The BRIEF parental questionnaire [86] contains 86 items to assess the subjective evaluation of executive functioning. It comprised eight sub-scales: (a) inhibit; (b) shift; (c) emotional control; (d) initiate; (e) working memory; (f) plan/organise; (g) organisation of materials; and (h) monitor.

### 2.3. Procedure

**Figure 1:**
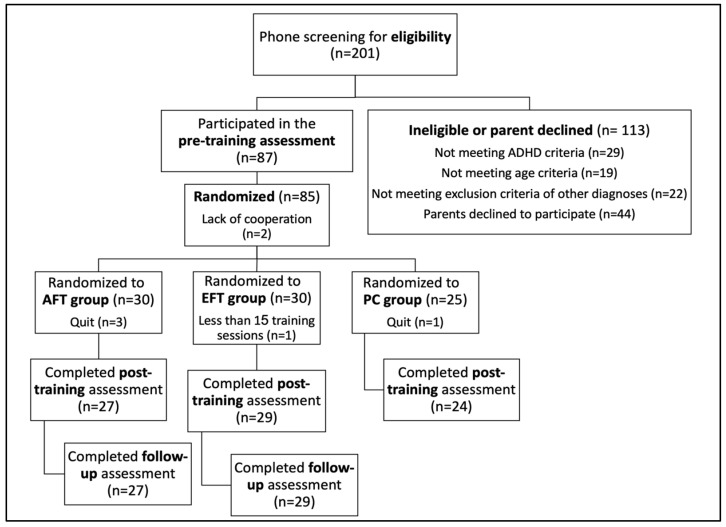
Recruitment and randomisation of the participants.

Participants were recruited based on the recommendation of professionals, such as paediatricians and school counsellors, and through flyers and ads on Facebook. Figure 1 illustrates the time flow of the study. Informed consent was obtained from all parents of participants. Participants were randomly assigned to one of three groups: an attention functions training (AFT) group, an executive functions training (EFT) group, and a passive control group (PC). Affiliation with the AFT and EFT groups was not known to the participants or their parents. To ensure effective blinding, both training protocols were designed with similar structures and appearances. The specific cognitive functions targeted by each task were embedded within engaging activities that were not readily distinguishable to participants, thus minimising the likelihood of inferring group allocation. Parents in both groups received identical information, being told that their children would participate in one of two training programs aimed at improving various functions in children with ADHD. To maintain blindness throughout the implementation process, trainers received training in the programs under the neutral designations of ‘Protocol 1’ and ‘Protocol 2’. Notably, the assessment research team was blind to the participants’ group affiliation as well. Each participant was assigned a unique subject number using a random number generator. Participants were included in the final analyses only if they had completed at least 15 out of 18 training sessions. The compliance criterion was met by all participants except for one participant from the EFT group.

All trainers were graduate students conducting their thesis research in the Attention Lab or participating in an advanced research seminar on attention. Prior to intervention implementation, trainers underwent comprehensive preparation that included hands-on experience with all training tasks and supervised practice sessions with a non-participant child. To ensure standardisation across sessions, trainers followed detailed protocols, including specific guidelines for each training program, general session management procedures, and a structured protocol for group activities. Each trainer conducted two daily sessions (one of each type). Sessions were conducted by teams of three trainers, enabling peer observation and mutual support. The first author attended most sessions to monitor implementation fidelity and maintain consistency across different training groups.

Participants were assessed individually by research assistants in the lab at baseline (i.e., before training = T1) and after completion of the training programs (i.e., after training = T2). In addition, approximately three months after completing the training programs, a follow-up assessment (i.e., T3) was conducted for participants in the two intervention groups to examine whether the results obtained at T2 had long-lasting effects. It is important to note that all three assessments were conducted after a 24 h medication washout period for participants treated with psychostimulants. All computer-based tasks were performed on a 24-inch LED monitor.

**Figure 2:**
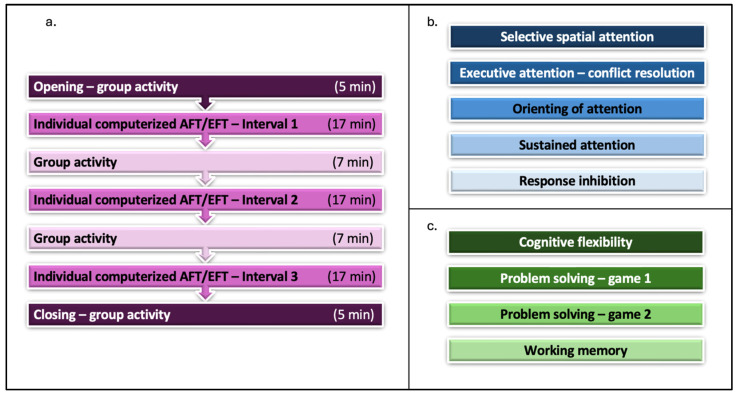
Training protocol structure and content. (**a**) The 75 min session structure, consisting of computerised training intervals and group activities. (**b**) Components of the computerised AFT protocol intervals. (**c**) Components of the computerised EFT protocol intervals.

The training protocols of the AFT and EFT groups had an identical structure. Each session lasted 75 min, and a total of 18 group sessions were held twice a week in a designated room at the university. Figure 2 illustrates the structure and content of the training protocols. The session structure (Figure 2a) consisted of three 17 min computerised training intervals interspersed with group activities. Each group was led by three qualified trainers and consisted of six to seven participants. Each session included an opening, three intervals of individual computerised training work with two breaks of group activities between, and a closing. The content of the computerised training work intervals was the only difference between the two protocols. The trainers were instructed to prompt the participants if they became distracted, to help them set feasible personal goals, and to help them monitor their progress. In addition, a novel group work module was used to promote new, meaningful social interactions among trainees. It included guidelines and activities for the opening, two breaks, and closing parts of the training sessions, in accordance with group developmental stages (i.e., forming, storming, norming, performing, and adjourning) [87,88].

The AFT group used two training tasks from the Cogmission software developed uniquely for this study: a Conjunctive Continuous Performance Task (CCPT, based on Shalev and colleagues [16]), designed to practice sustained attention, and a Go/No-Go task (based on Kolodny and colleagues [89]), designed to practice response inhibition. This group also used three training tasks from the Computerized Progressive Attention Training (CPAT) program [16]: (1) the Conjunctive Visual Search Task (based on Treisman and Gelade [80]), designed to improve selective-spatial attention; (2) the Combined Orienting and Flanker Task (based on Eriksen and Eriksen [90] and Posner and colleagues [91]), designed to improve orienting of attention; and (3) the Global–Local Task (based on Navon [92]), designed to improve executive attention and conflict resolution. Each of the tasks consists of a broad hierarchy of difficulty levels, and the transition between them occurs after consistent improvement is obtained and performance reaches a plateau at a given difficulty level [16].

The EFT group used a computerised version of the Set game, invented by Marsha Jean Falco in 1974 [93]. Set is a pattern-recognition card game where players must identify sets of three cards that share or differ in specific attributes, such as colour, shape, or number. It was used to train cognitive flexibility. In addition, this group used four computer games to train problem solving. Three of them were designed by Leo De Sol Games and were based on familiar games: (1) Rush Hour, a traffic-jam-themed game where players need to slide vehicles to clear a path for the red car to exit; (2) Pipes/Plumber, a puzzle game where players must connect pieces of pipe to create a functioning system; and (3) Sliding Puzzle, a traditional game where players must arrange pieces in the correct order by sliding them into the empty space on a grid. The fourth game, Thinkrolls Kings & Queens by Avokiddo, is a challenging adventure puzzle game that incorporates logic and physics, requiring players to navigate mazes and overcome obstacles using reasoning skills. Additionally, three games developed by Mindware Consulting Inc. were used to train working memory based on well-known paradigms in the field: Memory Racer, a fast-paced game based on the N-Back task [94], and two spatial memory tasks—Spatial Memory, requiring recall of locations on a grid, and Path Memory, which challenges players to remember and replicate a sequence of moves through a path.

This research was registered at ClinicalTrials.gov (identifier: NCT06657469).

### 2.4. Statistical Approach

Most analyses in the current study (i.e., ANOVAs, chi-squares, and *t*-tests) were carried out using SPSS v. 28 (IBM) software. Bayes Factor (BF) calculations were carried out using JASP v. 0.19.1 software. For all analyses, the threshold for statistical significance was set at *p* ≤ 0.05, and effect sizes are reported as partial eta squared (*η_p_*^2^) for ANOVAs and Cohen’s *d* for *t*-tests. In addition, all graphs include the standard error (SE) of each mean.

#### 2.4.1. Assessing the Effects of the Two Training Protocols

Differences between the three groups resulting from the two training protocols were tested using repeated measures ANOVAs with time of assessment (T1 vs. T2) as a within-subjects factor and group (AFT vs. EFT vs. PC) as a between-subjects factor. To evaluate long-lasting effects, further ANOVAs with repeated measures analyses were conducted, including assessments at three different time points (T1 vs. T2 vs. T3) as within-subjects factors and training group (AFT vs. EFT) as a between-subjects factor. The Bonferroni correction method was employed to correct for multiple comparisons in post hoc comparisons. In cases where no interaction between group and time was observed but a main effect of time was obtained, paired samples *t*-tests were performed within each group to assess differences between time points of assessment. In these instances, Bayesian statistics were also utilised, allowing for the testing of both the null and alternative hypotheses. The Bayes Factor (BF) provides a quantitative measure of the relative evidence in the data supporting one hypothesis over another. Specifically, the BF₁₀ is calculated as the ratio between the probability of the observed data under the alternative hypothesis (H₁) and its probability under the null hypothesis (H₀). In our analyses, the alternative hypothesis (H₁) always represented the expectation of improvement from T1 to T2. The interpretation of BF₁₀ values follows established guidelines: values supporting the alternative hypothesis (H₁): 1 < BF₁₀ < 3: weak evidence; 3 < BF₁₀ < 10: moderate evidence; and BF₁₀ > 10: strong evidence. Values supporting the null hypothesis (H₀): 1/3 < BF₁₀ < 1: weak evidence; 1/10 < BF₁₀ < 1/3: moderate evidence; and BF₁₀ < 1/10: strong evidence. Values close to 1 indicate insufficient evidence to support either hypothesis. This approach complements traditional null hypothesis significance testing by providing a measure of the strength of evidence for both the presence and absence of effects, rather than only evidence against the null hypothesis.

#### 2.4.2. Gender, Age, and Medication Use

Due to potential gender-related differences in some of the assessed measures, a chi-square test of independence was performed to examine the distribution of gender across groups (AFT vs. EFT vs. PC). No significant relationship was found between these variables, *χ*^2^_(2)_ = 1.544, *p* = 0.462, indicating that the distribution of gender did not differ by group. A one-way ANOVA was conducted to confirm the similarity of participants’ ages across the experimental groups. No significant difference (*F*_(2,77)_ = 0.11, *p* = 0.893, *η_p_*^2^ = 0.003) in age between the three groups was found. Additionally, a chi-square test of independence was also performed to determine if the distribution of participants’ medication use was different between the two training groups (i.e., AFT and EFT). No significant relationship was found between these variables, *χ*^2^_(1)_ = 0.001, *p* = 0.997, indicating that the distribution of medication intake did not differ between the two groups. Based on the above results, it was not necessary to control for age, gender, or medication use in all statistical analyses comparing the groups.

#### 2.4.3. Missing Data

One participant from the PC group did not complete the CPT at T2. Furthermore, the parents of one participant from the AFT group and one participant from the PC group did not complete the questionnaires at T2. These participants were only removed from the specific analyses involving the CPT and the questionnaires at T2.

#### 2.4.4. Data Trimming

Exclusion criteria were applied as follows: (a) omission error rate greater than 25% for the CPT (resulting in the exclusion of one outlier from the PC group); (b) average accuracy rate for all no-target trials < 80% in the CVST (no outliers were identified); (c) failure to correctly repeat a sequence of two blocks in the Corsi Block-Tapping Task Forward and Backward (resulting in the exclusion of four outliers: one from the AFT and one from the EFT groups for the Forward task, and one from the AFT and one from the PC groups for the Backward task); (d) identification of outliers using the 3*IQR method (three times the Interquartile Range (3*IQR stands for three times the Interquartile Range. The Interquartile Range (IQR) is a measure of statistical dispersion, specifically a measure of variability around the median, and it is calculated as the difference between the third quartile (Q3) and the first quartile (Q1))) for each measure in the CPM, the arithmetic task, and the questionnaires (resulting in the exclusion of two outliers from the arithmetic task—one from the AFT and one from the EFT groups).

## 3. Results

In analysing the effects of the AFT and EFT protocols, we distinguished between near and far transfer effects. Near transfer effects reflect improvements in tasks that directly assess one of the cognitive functions that were trained in a given protocol (e.g., improvement in sustained attention following attention training or improvement in working memory following executive functions training). Far transfer effects represent improvements in tasks or domains not directly trained but hypothesised to benefit from enhanced cognitive functioning (e.g., improvements in academic performance or behavioural symptoms following either attention or executive functions training).

### 3.1. Training Effects on Objective Measures of Attention Functioning and Executive Functioning

**Figure 3:**
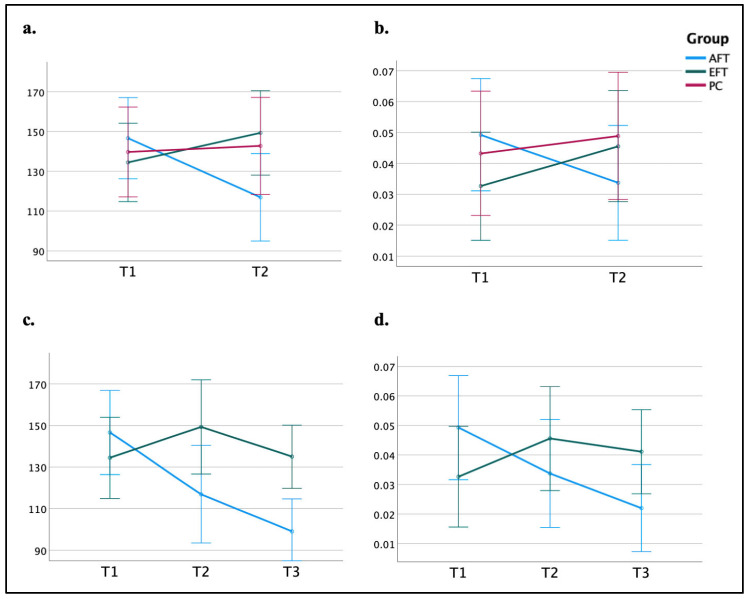
Sustained attention: performance across groups and testing sessions. (**a**) Standard deviation (SD) of reaction time (RT) in the CPT, as a function of time (T1, T2) and group (AFT, EFT, PC). (**b**) Omission error rate in the CPT, as a function of time (T1, T2) and group (AFT, EFT, PC). (**c**) Standard deviation (SD) of reaction time (RT) in the CPT, as a function of time (T1, T2, T3) and group (AFT, EFT). (**d**) Omission error rate in the CPT, as a function of time (T1, T2, T3) and group (AFT, EFT). Error bars represent the standard error of the mean (SEM).

Sustained attention was measured using two measures derived from the CPT: (a) the SD of RT, and (b) the omission error rate. A two-way repeated measures ANOVA conducted on the SD of RT revealed a significant interaction of Time × Group (*F*_(2,75)_ = 6.26, *p* = 0.003, *η_p_*^2^ = 0.143), as can be seen in Figure 3a. Bonferroni post hoc tests showed that participants in the AFT group demonstrated improved response consistency, with the SD of RT decreasing by 30 ms from T1 (*M* = 147, *SD* = 64) to T2 (*M* = 117, *SD* = 47; *p* = 0.03, Cohen’s *d* = 0.53). No significant changes were measured in the other two groups. A similar two-way repeated measures ANOVA on the omission error rate revealed no interaction of Time × Group (*F*_(2,75)_ = 2.37, *p* = 0.10, *η_p_*^2^ = 0.059).

Repeating the above-mentioned analyses with three time-points (T1 vs. T2 vs. T3) and two groups (AFT vs. EFT) revealed significant interactions of Time × Group for both the SD of RT (*F*_(2,54)_ = 9.56, *p* < 0.001, *η_p_*^2^ = 0.15; see Figure 3c) and the omission error rate (*F*_(2,54)_ = 4.72, *p* = 0.011, *η_p_*^2^ = 0.08; see Figure 3d). Bonferroni post hoc tests showed that the AFT group demonstrated progressive improvements in response consistency: the SD of RT decreased by 30 ms from T1 (*M* = 147, *SD* = 64) to T2 (*M* = 117, *SD* = 47; *p* = 0.014, Cohen’s *d* = 0.57) and decreased by 48 ms at T3 (*M* = 99, *SD* = 37; *p* < 0.001, Cohen’s *d* = 0.91 compared to T1). Furthermore, enhanced sustained attention of the AFT group was also found in reduced omission errors, with a significant decrease of 2.7% from T1 (*M* = 0.049, *SD* = 0.056) to T3 (M = 0.022, SD = 0.030; *p* = 0.012, Cohen’s *d* = 0.62). In the EFT group, there were no significant differences in the SD of RT and the omission rate between the different time points (all *p*s > 0.169). Taken together, these results indicate that participants in the AFT group considerably improved their ability to sustain attention over a long period of time while performing a monotonous task at T2 compared to T1. Importantly, this effect was even more pronounced in the follow-up, approximately three months after the end of the training. Furthermore, these improvements were large and unique to the AFT group.

Selective-spatial attention was measured using the CVST’s mean RTs and the accuracy rates of all eight task conditions (i.e., 4/8/16/32 items’ display sizes with and without a target). A four-way repeated measures ANOVA was conducted on the mean RTs with time (T1 vs. T2), the target’s presence (target present vs. target absent), and the set size (4 vs. 8 vs. 16 vs. 32) as within-subject factors, and group (AFT vs. EFT vs. PC) as a between-subjects factor. The analysis revealed a significant interaction among Time × Target’s presence × Set size × Group (*F*_(5.35, 206.07)_ = 2.76, *p* = 0.017, *η_p_*^2^ = 0.067). To further investigate the interaction source, we calculated the difference in mean RTs between T1 and T2 (i.e., T1 minus T2) separately for each of the eight conditions. Two-way repeated measures ANOVAs were carried out separately for displays with and without target, with set size as a within-subjects factor and group as a between-subjects factor.

**Figure 4:**
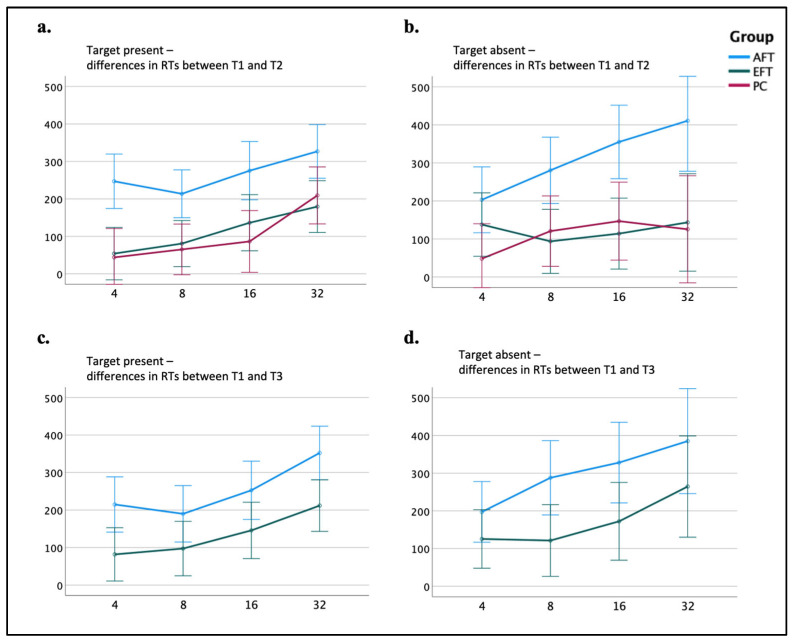
Selective-spatial attention: performance in the CVST by group and testing session. (**a**) Differences in RTs between T1 and T2 of the ‘target present’ displays as a function group (AFT, EFT, PC). (**b**) Differences in RTs between T1 and T2 of the ‘target absent’ displays as a function group (AFT, EFT, PC). (**c**) Differences in RTs between T1 and T3 of the ‘target present’ displays as a function group (AFT, EFT). (**d**) Differences in RTs between T1 and T3 of the ‘target absent’ displays as a function group (AFT, EFT). Error bars represent the standard error of the mean (SEM).

For ‘target present’ displays, a main effect of group was found (*F*_(2,77)_ = 9.59, *p* < 0.001, *η_p_*^2^ = 0.199), suggesting that the improvement in RTs of the AFT group in 266 ms (*SD* = 166) was significantly greater than the EFT group’s improvement of 113 ms (*SD* = 145; *p* = 0.001, Cohen’s *d* = 0.82) and the PC group’s improvement of 101 ms (*SD* = 147; *p* < 0.001, Cohen’s *d* = 0.88). For ‘target absent’ displays, a marginally significant interaction between set size and group was found (*F*_(4.67,179.68)_ = 2.82, *p* = 0.053, *η_p_*^2^ = 0.056). Bonferroni post hoc tests showed that the improvements in RTs of the AFT group were significantly greater than those in the EFT group for set sizes of 8, 16, and 32 (all *p*s < 0.015), but not for a set size of 4, and significantly greater than those of the PC group for all set sizes (all *p*s < 0.049) (see Figure 4 for the results of both two-way repeated measures ANOVAs). The parallel analysis of four-way repeated measures ANOVA on the accuracy rates resulted in no significant interaction of Time × Group. It is important to note that the accuracy rates of all three groups were high to begin with during T1 (see Appendix A for descriptive statistics). Taken together, these findings indicate that the AFT group showed greater improvement in RTs at T2 compared to T1 than the EFT and the PC groups with no significant changes in accuracy. Namely, the improvement in RT was not compromised by a decrease in accuracy. Therefore, this improvement reflects enhanced efficiency in focusing on a restricted spatial area effectively, suppressing adjacent irrelevant information.

We used the CVST’s T3 results to examine if the results achieved by the AFT group in selective-spatial attention during T2 had a long-lasting effect. This time, we calculated the differences in RTs between T1 and T3 separately for each one of the eight conditions. For the ‘target present’ displays, a main effect of group was found (*F*_(1,54)_ = 7.09, *p* = 0.010, *η_p_*^2^ = 0.116), suggesting that the difference in RTs of the AFT group (*M* = 252, *SD* = 190) was significantly greater than that of the EFT group (*M* = 134, *SD* = 141). For the ‘target absent’ displays, similar results were obtained, including a main effect of group (*F*_(1,54)_ = 4.71, *p* = 0.034, *η_p_*^2^ = 0.080), also indicating that the difference in RTs of the AFT group (*M* = 299, *SD* = 232) was significantly greater than that of the EFT group (*M* = 171, *SD* = 212). Therefore, the comparisons between T1 and T3 replicated the results obtained when comparing T1 to T2, indicating that the AFT’s improved performance in this task has a long-lasting effect (see Figure 4).

Visuospatial working memory capacity was measured with the Corsi Block-Tapping Task Forward and Backward. Two two-way repeated measures ANOVAs were conducted on the summary scores of both tasks. The analyses did not reveal significant interactions between Time × Group (both Fs < 1.67; both *p*s > 0.196). In addition, in the analysis of the Corsi Block Backward, there was no main effect of time, whereas in the Corsi Block Forward, a main effect of time was found (*F*_(1,75)_ = 9.67, *p* = 0.003, *η_p_*^2^ = 0.114), suggesting that all groups performed better at T2 (*M* = 42.26, *SD* = 18.00) compared to T1 (*M* = 37.04, *SD* = 15.59), showing an overall mean improvement of 5.22 points (see Appendix B for descriptive statistics). Therefore, we conducted paired samples *t*-tests to separately estimate the changes between T1 and T2 for each group. For the AFT and PC groups, no significant differences were found (*t*_(25)_ = −1.47, *p* = 0.154, Cohen’s *d* = 0.29; *t*_(23)_ = −1.87, *p* = 0.074, Cohen’s *d* = 0.38, respectively), whereas in the EFT group a significant improvement in T2 (*M* = 41.4, *SD* = 20.4) compared to T1 (*M* = 35.8, *SD* = 19.0), representing a mean improvement of 5.6 points, was obtained (*t*_(27)_ = −2.08, *p* = 0.048, Cohen’s *d* = 0.39). Nevertheless, the Bayesian factor for this *t*-test was close to 1 (BF_10_ = 1.23), indicating that the data do not provide sufficient evidence for H1 or for H0. Based on the results of the *t*-tests and the Bayesian factor value, it appears that neither of the groups demonstrated a substantial improvement in working memory at T2.

**Figure 5:**
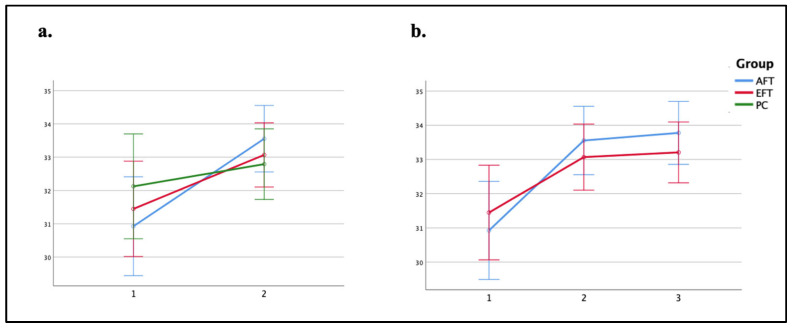
Nonverbal abstract reasoning: performance in the Raven’s Colored Progressive Matrices (CPM) by group and testing session. (**a**) Total raw score in the CPM, as a function of time (T1, T2) and group (AFT, EFT, PC). (**b**) Total raw score in the CPM, as a function of time (T1, T2, T3) and group (AFT, EFT). Error bars represent the standard error of the mean (SEM).

To assess nonverbal abstract reasoning, the total raw score of the Raven’s Colored Progressive Matrices (CPM) was used. Figure 5 presents the performance on the CPM by group and time of assessment. A two-way repeated measures ANOVA revealed a significant interaction effect of Time × Group (*F*_(2,77)_ = 3.30, *p* = 0.043, *η_p_*^2^ = 0.078). Bonferroni post hoc tests indicated that participants in the AFT and EFT groups exhibited improved performance on this task at T2 (*M* = 33.56, *SD* = 2.52; *M* = 33.07, *SD* = 2.67, respectively) compared to T1 (*M* = 30.93, *SD* = 4.37; improvement of 2.63 points, *p* < 0.001, Cohen’s *d* = −0.80; *M* = 31.45, *SD* = 3.01; improvement of 1.62 points, *p* = 0.002, Cohen’s *d* = −0.49, respectively). However, in the PC group, the change was not significant (T1: *M* = 32.13, *SD* = 4.15; T2: *M* = 32.79, *SD* = 2.84; improvement of 0.66 points, *p* = 0.236).

To assess if the improvements in both training groups were maintained over time, a similar analysis was conducted with three-time points (T1 vs. T2 vs. T3) and two groups (AFT vs. EFT). This analysis yielded a significant main effect of time (*F*_(1.72, 92.68)_ = 32.24, *p* < 0.001, *η_p_*^2^ = 0.374), indicating that both the AFT and EFT groups exhibited enhanced performance at T2 (*M* = 33.30, *SD* = 2.59; improvement of 2.1 points from T1) and T3 (*M* = 33.48, *SD* = 2.39; improvement of 2.28 points from T1) compared to T1 (*M* = 31.20, *SD* = 3.70; *p* < 0.001, Cohen’s *d* = −0.72; *p* < 0.0001, Cohen’s d = −0.78, respectively). These findings suggest that the robust improvements achieved at T2 were maintained at T3 as well, as can be seen in Figure 5.

Taken together, the objective measures of attention functioning and executive functioning revealed clear differences between the AFT and EFT protocols. The AFT protocol resulted in robust improvements in sustained attention (mean reduction of 48 ms in SD of RT and 2.7% reduction in omission errors rate) between T1 and T3 vs. no significant change for EFT and selective-spatial attention (mean RT improvement of 266 ms for AFT vs. 113 ms for EFT, *p* = 0.001, Cohen’s *d* = 0.82). These improvements were maintained at a three-month follow-up. In contrast, while both protocols enhanced nonverbal abstract reasoning (improvement of 2.63 points for AFT, *p* < 0.001, Cohen’s *d* = −0.80; improvement of 1.62 points for EFT, *p* = 0.002, Cohen’s *d* = −0.49), neither produced significant improvements in working memory performance (all *p*s > 0.169).

### 3.2. Training Effects on Subjective Measures of Attention Functioning and Executive Functioning

**Figure 6:**
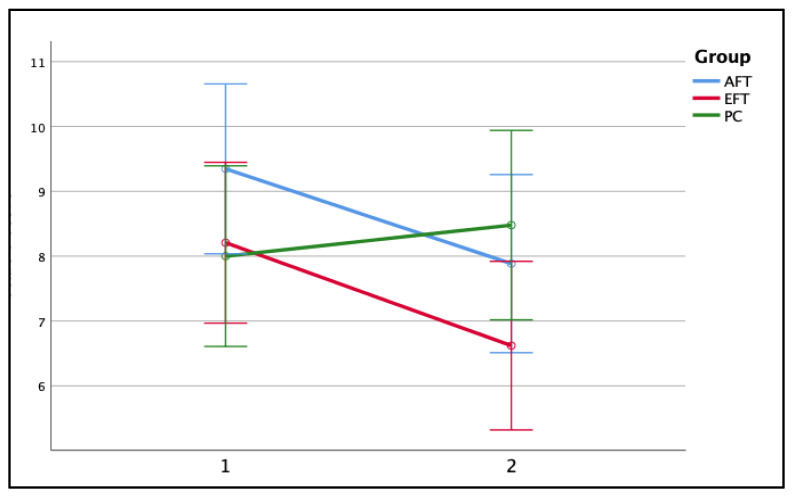
CBCL attention problems syndrome sub-scale’s score as a function of time (T1, T2) and group (AFT, EFT, PC). Error bars represent the standard error of the mean (SEM).

To assess inattention symptomatology, we conducted a two-way repeated measures ANOVA using the ‘attention problems’ sub-scale score of the CBCL questionnaire. This analysis resulted in an interaction between Time and Group (*F*_(2,75)_ = 4.94, *p* = 0.010, *η_p_^2^* = 0.116), as shown in Figure 6. Bonferroni post hoc tests indicated that participants in the AFT and EFT groups showed a significant decrease in ADHD inattention symptoms as evaluated by parents at T2 (*M* = 7.88, *SD* = 3.08; *M* = 6.62, *SD* = 3.58, respectively) compared to T1 (*M* = 9.35, *SD* = 3.51; reduction of 1.47 points, indicating a 15.7% improvement, *p* < 0.005, Cohen’s *d* = 0.43; *M* = 8.21, *SD* = 3.21; reduction of 1.59 points, indicating a 19.4% improvement, *p* = 0.001, Cohen’s *d* = 0.46, respectively). In the PC group, there was no significant change between T2 (*M* = 8.48, *SD* = 3.84) compared to T1 (*M* = 8.00, *SD* = 3.30; increase of 0.48 points, *p* = 0.376), indicating that the improvements in attention problems were specific to the training interventions.

In addition, three raw scores from the CBCL questionnaire were used to assess adaptive and maladaptive behaviour: (a) internalising behaviour score (comprising the anxious/depressed, withdrawn/depressed, and the somatic complaints syndrome sub-scales); (b) externalising behaviour score (comprising the rule-breaking behaviour, and the aggressive behaviour syndrome sub-scales); and (c) social problems syndrome sub-scale’s score. Three two-way repeated measures ANOVAs on each one of these raw scores were conducted. All analyses yielded similar results, with no Time × Group interaction. Appendix C provides the descriptive statistics and analyses results.

Eight two-way repeated measures ANOVAs were conducted on each one of the BRIEF questionnaire’s sub-scales’ raw scores, as a subjective measurement of executive functioning. All analyses yielded similar results: no interaction between Time and Group and no main effect of time (all Fs < 1.08; all *p*s > 0.343). See Appendix D for descriptive statistics.

### 3.3. Training Effects on Arithmetic Performance

A couple of two-way repeated measures ANOVAs on the task duration and on the accuracy of the arithmetic task were conducted. Both analyses revealed no significant interaction of Time × Group (both Fs < 1.51; both *p*s > 0.228), yet a significant main effect of time was found in both analyses, (F(1,75) = 9.81, *p* = 0.002, *η_p_^2^* = 0.116; F(1,75) = 9.72, *p* = 0.003, *η_p_*^2^ = 0.115, respectively), suggesting that all three groups performed better at Time 2, compared to Time 1 (See Appendix E for descriptive statistics). Consequently, we conducted paired samples *t*-tests, for both measures, to estimate the changes between T1 and T2, separately for each group. For the AFT group, a significant difference in task duration (*t*_(25)_ = 2.61, *p* = 0.015, Cohen’s *d* = 0.51) was found, due to a faster performance at T2 (*M* = 440 sec, *SD* = 243) compared to T1 (*M* = 528 sec, *SD* = 284), demonstrating a reduction of 88 s in completion time. Additionally, a significant difference in accuracy (*t*_(25)_ = −2.38, *p* = 0.025, Cohen’s *d* = 0.47), due to a higher accuracy at T2 (*M* = 88.56%, *SD* = 16.99) compared to T1 (*M* = 84.62%, *SD* = 18.86), reflecting an improvement of 3.94 percentage points. The Bayesian factors of these *t*-tests were greater than 3 (BF_10_ = 6.58; BF_10_ = 4.27, respectively), indicating substantial support for H1. For the other two groups, no significant improvements at T2 compared to T1 were found (all *p*s > 0.060). Taken together, based on the results of the *t*-tests and Bayesian factor values, it appears that only the AFT group demonstrated a notable improvement in the arithmetic performance between T1 and T2.

Repeating the separate two-way repeated measures ANOVAs analyses with three time points (T1 vs. T2 vs. T3) and two groups (AFT vs. EFT) yielded similar results, namely no interactions of Time × Group (both Fs < 2.11; both *p*s > 0.127), only main effects were obtained for task duration: *F*_(2,104)_ = 4.08, *p* = 0.020, *η_p_*^2^ = 0.073, and for accuracy: *F*_(2,104)_ = 3.80, *p* = 0.025, *η_p_*^2^ = 0.068). To test whether the trends observed between T1 and T2, as measured by paired samples *t*-tests, were replicated at T3, we repeated them by comparing T1 to T3. None of the comparisons reached significance (all *p*s > 0.060).

### 3.4. Summary of the Effects of the AFT and the EFT Protocols

To summarise the effects of the AFT and the EFT protocols, the results of the various outcome measures are presented in Table 3. ‘Near transfer’ refers to improvements in tasks that are similar or directly related to the specific skills trained during the intervention. All other outcomes were classified as ‘far transfer’ (i.e., improvements in tasks that are different from the specific skills trained, indicating a broader generalisation of the training’s effects to other cognitive abilities, behavioural symptoms, or academic performance. Additionally, the plus and minus signs in the table indicate whether long-lasting of improvements were documented, as measured in the follow-up assessment.

## 4. Discussion

ADHD is a prevalent developmental disorder with substantial negative effects across various life domains. While treatments, such as stimulant medications and behavioural therapy, are widely used, their efficacy in addressing core cognitive deficits remains limited. Moreover, previous research on neurocognitive training as an alternative treatment for ADHD has produced inconsistent results, with open questions regarding the transferability of cognitive improvements to real-life functioning and the long-term sustainability of these effects. This study aimed to address these gaps by rigorously evaluating two theory-driven, computer-based neurocognitive small-group training programs for children with ADHD. These programs were designed based on the attention functions model [42] and theories that emphasise executive functions (EFs) as the core deficit of ADHD [47,53,54]. We created parallel intervention protocols targeting attention and EFs while carefully controlling for certain factors, such as engagement, motivation, and contact with trainers, to ensure that any observed effects could be attributed to the interventions themselves. The results of this study provide new insights into the efficacy of neurocognitive small-group training programs in improving cognitive functions in children with ADHD.

Our results demonstrate a significant, unique improvement in sustained attention following training with the AFT protocol, which was maintained in the follow-up assessment. The current study used the Cogmission Version 3.0 Beta software to train sustained attention, and the findings are consistent with some of the previous studies that demonstrated improved sustained attention after training with the CPAT [15,76,95,96]. Studies that implemented other attention trainings manifested mixed results, as some reported no improvement in sustained attention [31,97], whereas Lange and colleagues [98] reported an improvement in vigilance after training. Nevertheless, the latter can be considered an improvement in response inhibition as the only recorded improvement was in commission errors.

The AFT group also presented with faster performance in selective-spatial attention (as measured by the CVST) following training compared to the other two groups. Importantly, no changes in accuracy rates were recorded in any of the three groups, suggesting that the AFT protocol may have caused participants to be more efficient in selective-spatial attention, as the improvement in response times was not a result of a trade-off between speed and accuracy. The improved speed of reactions was replicated in the follow-up assessment and provided another indication of the long-lasting effect of the AFT protocol. Improved selective-spatial attention was also reported in previous studies with higher education students and children with foetal alcohol spectrum disorder after training with the CPAT [15,95]. It can be concluded that our results demonstrate unique gains that can be attributed to the specific content of the AFT protocol, as reflected by improvements in both attention assessment tasks. Notably, the assessment tasks differed from the training tasks. Hence, the data of the AFT group clearly represent unique near transfer effects of the trained cognitive mechanisms. Importantly, these unique near transfer effects were maintained at follow-up, indicating long-term effects of the AFT protocol.

In light of previous studies presenting improved EFs after compatible cognitive training, it was reasonable to hypothesise that our EFT protocol would yield positive results manifested in objective measures of EFs. However, the EFT group did not show a greater improvement in visuospatial working memory compared to the AFT and PC groups, although it was specifically trained as part of its protocol. Nonetheless, our results do not contradict previous studies that reported improved working memory after training (see for example, [28,29,30]), as our EFT protocol focused not only on WM. Therefore, it could be argued that the intensiveness in which each EF’s component was trained was lower in our protocol. A meta-analysis of training programs for children with ADHD supports this explanation, showing that intensive short-term memory training led to moderate improvements, while less intensive training of attention and mixed EFs showed minimal effects [37].

As for nonverbal abstract reasoning, our results indicated that although the PC group performed similarly at the pre- and post-training assessments, both intervention groups showed significant improvement following training. The results of the intervention groups are consistent with previous studies that demonstrated improved nonverbal abstract reasoning after working memory training or attention training [17,28,29,99,100,101]. Importantly, in the present study, the results were maintained in the follow-up assessment, indicating the long-lasting effect of both protocols and pointing out that both training protocols are beneficial for nonverbal abstract reasoning. Nevertheless, it is essential to emphasise the similarities between the training tasks of the EFT group and the Raven’s test. While the Raven’s items require the identification of a missing element to complete a pattern, some training tasks were also based on visual puzzles, in which participants were asked to identify the correct location or direction of different pieces in the pattern to solve it. Moreover, the EFT protocol used the Set game, where participants are required to analyse different visual domains simultaneously, as needed in the Raven. Another EFT component that should be considered in this context is the n-back training task, as a meta-analysis of n-back training programs with healthy adult participants concluded that training significantly positively affected nonverbal abstract reasoning [102]. Accordingly, the EFT results should be considered a near transfer effect, whereas it can be considered a far transfer effect for the AFT group.

Concerning the behavioural symptoms of ADHD, results indicated that parents in both training groups reported a decreased level of attention problems following training, whereas parents of children in the PC group did not report any changes. The lack of improvement following training in the other CBCL child behaviour sub-scales, although intermixed, should be emphasised, as it rules out the expectancy effect (i.e., changes in behaviour that result from participants’ or parents’ expectations rather than the actual effects of the intervention itself). Taken together, these results indicate a far transfer effect of both training protocols and imply that both are beneficial for behavioural symptoms. A possible explanation relies on the fact that inattention symptoms can be associated with the content of the cognitive components of both training protocols. An alternative explanation lies in our novel ‘small group work’ module, which includes activities requiring resourceful thinking and enables them to learn from their peers how to cope with different situations. The unique role description of the trainers could provide another possible explanation, as they might have raised awareness by prompting the trainees whenever they became distracted. Similarly to our findings, several previous studies reported reduced ADHD symptomatology after training compared to a control group [16,31,33,103]. However, a closer look at their results reveals that the expectancy effect could not be ruled out. The one exception is Shalev and colleagues [16], who demonstrated a decreased level of ADHD symptoms after attention training compared to an active control group, rated by blind observers.

The results of the BRIEF’s parental questionnaire sub-scales indicated that no improvement in subjective evaluation of participants’ EFs occurred in the EFT or the AFT groups. This was somewhat surprising, as it could have been argued that the parents should have expected improvements in areas like the ones that are addressed in the BRIEF questionnaire, especially because they can easily be related to behavioural symptoms of ADHD. This lack of improvement further supports the specificity of the observed improvement in ADHD symptoms that was recorded in the CBCL’s attention problems sub-scale, suggesting, once again that it may be attributed to the training protocols. Previous cognitive training studies that also applied the BRIEF questionnaire presented inconsistent findings [97,103].

With respect to arithmetic performance, we hypothesised that both training protocols would be beneficial. The results somewhat supported this hypothesis, in that the performance of the AFT group showed some improvement after the training, demonstrating faster and more accurate arithmetic performance compared to before. While this tentatively suggests that the AFT protocol may have positive carryover effects to untrained academic areas (i.e., far transfer), the lack of an interaction effect precludes strong conclusions. No effect on arithmetic performance was found in the EFT group. Taken together, our findings are not in line with the meta-analysis by Rapport and colleagues [37] which concluded that neurocognitive training has no positive effect on academic achievements, regardless of the trained cognitive functions. One important question is why the improvement in arithmetic was not persistent as were the improvements in attention and in nonverbal abstract reasoning. A possible explanation is that the sustainability of improvements in domain-general abilities and domain-specific abilities may differ. Domain-general cognitive abilities, such as abstract reasoning, may benefit more directly and sustainably from enhanced attentional functioning, as both rely on similar underlying neural networks supporting general cognitive processing. In contrast, arithmetic performance, being a domain-specific skill, likely requires ongoing practice and explicit instruction in addition to improved attentional resources. While enhanced attention can support mathematical computations through better focus and reduced cognitive load, maintaining gains in domain-specific academic skills may require the continued integration of improved attentional functions with subject-specific practice. This pattern suggests that while attention training can provide cognitive support for academic performance, sustained improvement in domain-specific skills might require complementary academic instruction to consolidate these gains.

The present AFT protocol consistently yielded robust near transfer effects on sustained attention and selective-spatial attention. Moreover, this protocol produced several encouraging far transfer effects pertaining to different aspects of everyday functioning: behavioural (reduced ADHD symptoms), cognitive (improved performance on nonverbal abstract reasoning test), and academic (enhanced arithmetic performance). One of the unique characteristics underlying the AFT protocol’s effectiveness is that the trained attentional functions are basic cognitive functions, unlike the EFT protocol, which addresses more advanced cognitive functions. Such higher-order cognitive functions rely on a variety of basic cognitive functions; hence, the EFT protocol may have been less focused compared to the AFT protocol, which may explain its attenuated efficiency in reinforcing direct links between performance on each training activity and specific cognitive mechanisms.

### 4.1. Limitations and Future Research

As mentioned above, the current study was designed to address several essential criticisms within the neurocognitive training field; however, it has several limitations that warrant consideration. The EFT protocol’s content was purposefully designed to tackle a key criticism regarding previous cognitive training studies (i.e., involving simple, non-progressive training protocols for the active control group). However, the challenge lies in detecting differential effects between the two training protocols given their highly similar content. This similarity makes it difficult to isolate the unique contributions of each protocol, which may limit our ability to identify distinct effects. A second limitation pertains to the number of participants in each group (slightly under 30 participants), contributing to limited statistical power. Despite recruiting approximately 200 potential families, over 50% did not meet our inclusion criteria or declined participation (e.g., families residing far from the university campus or with children engaged in many extracurricular activities). Another limitation we encountered from the outset was the difficulty in recruiting teachers to complete behavioural symptom and academic performance questionnaires intended for use in the study.

As for future research directions, isolating the influence of the small-group work module on the outcome measures would be insightful, as this module was incorporated into both training programs, precluding an examination of its unique contribution. Another important future research direction would be to compare the results of the AFT protocol to an upgraded protocol that also encompasses an academic skill-focused component or a training program solely focused on that specific academic skill without neurocognitive training. Lastly, when investigating the influence of the AFT and EFT protocols with larger samples, an important future research direction would be to analyse the recorded improvements in relation to participants’ individual characteristics. Specifically, examining how baseline attentional/cognitive profiles, age, ADHD subtypes, and comorbid conditions might moderate training outcomes could reveal important individual difference factors. Such an analysis could shed light on which participant is most likely to benefit from each type of training protocol, potentially leading to more personalised intervention approaches.

### 4.2. Implications

The promising effects of the AFT protocol suggest that it is a neurocognitive training intervention that holds great potential for children with ADHD. Several key elements appear to contribute to its efficacy, including the fact that it is (a) theory-driven, (b) focused on a small number of basic cognitive functions, (c) structured, (d) intensive, (e) gradually increasing in difficulty level, (f) personally adjusted according to the trainee’s abilities, and (g) incorporating feedback addressing both cognitive and socio-emotional needs of trainees. If appropriately addressed, these key elements could serve as the foundation for successful interventions targeting children with various cognitive impairments and therefore could be implemented by clinicians and professionals, regardless of their specific training in executing the AFT protocol. Clinicians and professionals could utilise these key elements as a ‘recipe’ for successful neurocognitive training. Alternatively, they could integrate any of these ‘ingredients’ to enhance their existing non-pharmacological treatment methods.

## Figures and Tables

**Table 1:** Distribution of gender, age, and medication status across groups.

	Age			Gender		Medication Status	
Group	*n*	*M*	*SD*		*n*		*n*
AFT	27	10.38	1.12	Boys	18	Use	12
				Girls	9	Do Not Use	15
EFT	29	10.46	1.07	Boys	16	Use	13
				Girls	13	Do Not Use	16
PC	24	10.53	1.15	Boys	17	Use	19
				Girls	7	Do Not Use	5

**Table 2:** Overview of assessment tools and outcome measures.

Domain	Assessment Tool	Description	Outcome Measures
Sustained attention	Continuous Performance Test (CPT)	Participants respond to target geometric shapes while ignoring others	SD of response times Omission errors
Selective-spatial attention	Conjunctive Visual Search Task (CVST)	Search for blue square among red squares and blue circles	Response times Accuracy rates
Visuospatial working memory	Corsi Block-Tapping Task Forward	Reproduce sequence of lit blocks in same order	Summary score (longest sequence × correct sequences)
Visuospatial working memory	Corsi Block-Tapping Task Backward	Reproduce sequence of lit blocks in reverse order	Summary score (longest sequence × correct sequences)
Nonverbal abstract reasoning	Raven’s Colored Progressive Matrices	Complete visual patterns	Raw score
Behavioural symptoms	CBCL Questionnaire	Parent-rated behavioural assessment	Attention problems Other syndrome scales
Executive functioning	BRIEF Questionnaire	Parent-rated executive function assessment	Eight sub-scale scores
Arithmetic	Arithmetic Evaluation	Basic arithmetic operations	Speed (completion time) Accuracy (% correct)

**Table 3:** Outcome measures that produced significant improvements.

	AFT	EFT
Near transfer	Sustained attention (+)	Nonverbal abstract reasoning (+)
	Selective-spatial attention (+)	
Far transfer	ADHD symptomatology (N/A)	ADHD symptomatology (N/A)
	Nonverbal abstract reasoning (+)	
	Arithmetic performance ^1^ (−)	

Notes. ^1^ The interaction effect was not significant. Paired samples *t*-tests yielded significant results. (+) Significant improvement at T3 compared to T1 *(p* < 0.05). (−) No significant improvement at T3 compared to T1. (N/A) Not assessed at T3.

## Data Availability

The datasets supporting the reported results are available upon reasonable request.

## Appendix A

**Table A1:** Means and standard deviations of average RTs and accuracy rates in the CVST for all groups in all eight conditions at Time 1, Time 2, and Time 3.

Time	Target’s Presence	Set Size	Group	Average RTs (ms)		Accuracy Rates		*n*
				*M*	*SD*	*M*	*SD*	
1	Target present	4	AFT	956	188	0.974	0.035	27
			EFT	933	191	0.969	0.049	29
			PC	929	194	0.945	0.056	24
		8	AFT	954	214	0.970	0.049	27
			EFT	963	187	0.944	0.068	29
			PC	958	185	0.973	0.033	24
		16	AFT	1110	291	0.920	0.088	27
			EFT	1108	257	0.922	0.075	29
			PC	1086	230	0.935	0.072	24
		32	AFT	1284	280	0.868	0.126	27
			EFT	1261	266	0.886	0.093	29
			PC	1286	274	0.842	0.102	24
	Target absent	4	AFT	1068	248	0.985	0.027	27
			EFT	1129	267	0.961	0.056	29
			PC	1087	244	0.971	0.046	24
		8	AFT	1172	336	0.985	0.023	27
			EFT	1149	269	0.976	0.047	29
			PC	1141	249	0.969	0.060	24
		16	AFT	1384	459	0.990	0.020	27
			EFT	1312	291	0.982	0.034	29
			PC	1267	307	0.959	0.066	24
		32	AFT	1666	596	0.987	0.033	27
			EFT	1646	425	0.984	0.028	29
			PC	1548	422	0.974	0.035	24
2	Target present	4	AFT	709	188	0.972	0.052	27
			EFT	879	263	0.959	0.047	29
			PC	884	194	0.949	0.071	24
		8	AFT	740	208	0.978	0.042	27
			EFT	882	194	0.970	0.039	29
			PC	893	191	0.964	0.045	24
		16	AFT	834	214	0.937	0.055	27
			EFT	972	205	0.938	0.061	29
			PC	1000	198	0.939	0.051	24
		32	AFT	957	247	0.894	0.089	27
			EFT	1082	234	0.921	0.108	29
			PC	1076	224	0.917	0.075	24
	Target absent	4	AFT	865	275	0.964	0.048	27
			EFT	991	244	0.977	0.039	29
			PC	1039	296	0.962	0.048	24
		8	AFT	892	301	0.980	0.038	27
			EFT	1055	323	0.988	0.029	29
			PC	1020	197	0.968	0.050	24
		16	AFT	1028	322	0.989	0.021	27
			EFT	1198	265	0.977	0.032	29
			PC	1120	242	0.973	0.059	24
		32	AFT	1255	403	0.983	0.044	27
			EFT	1503	433	0.977	0.039	29
			PC	1422	454	0.967	0.053	24
3	Displays with target	4	AFT	741	208	0.961	0.042	27
			EFT	851	213	0.967	0.036	29
		8	AFT	764	224	0.976	0.045	27
			EFT	866	232	0.973	0.033	29
		16	AFT	857	225	0.950	0.060	27
			EFT	963	279	0.926	0.059	29
		32	AFT	933	226	0.928	0.092	27
			EFT	1049	258	0.913	0.081	29
	Displays with no target	4	AFT	871	256	0.961	0.047	27
			EFT	1004	254	0.980	0.038	29
		8	AFT	885	243	0.983	0.034	27
			EFT	1027	283	0.964	0.065	29
		16	AFT	1056	360	0.983	0.039	27
			EFT	1140	282	0.979	0.037	29
		32	AFT	1281	405	0.985	0.036	27
			EFT	1382	394	0.968	0.049	29

## Appendix B

**Table A2:** Means and standard deviations of the summary scores of the working memory tasks for all groups at Time 1 and Time 2.

Time	Group	Summary Scores of the Tasks					
		Corsi Block-Tapping Task Forward			Corsi Block-Tapping Task Backward		
		*M*	*SD*	*n*	*M*	*SD*	*n*
1	AFT	40.23	15.41	26	38.58	17.91	26
	EFT	35.82	18.99	28	35.17	15.97	29
	PC	35.00	10.72	24	44.04	10.78	23
2	AFT	44.69	16.60	26	38.50	16.08	26
	EFT	41.43	20.43	28	39.14	14.37	29
	PC	40.58	16.89	24	38.35	14.56	23

## Appendix C

**Table A3:** Means and standard deviations of the internalising behaviour, the externalising behaviour, and the social problems syndrome sub-scale of the CBCL for all groups at Time 1 and Time 2.

Time	Group	Internalising Behaviour Score		Externalising Behaviour Score		Social Problems Score		*n*
		*M*	*SD*	*M*	*SD*	*M*	*SD*	
1	AFT	11.92	8.35	9.04	6.80	4.73	3.70	26
	EFT	9.72	7.05	10.00	7.27	4.14	3.71	29
	PC	10.26	6.75	12.52	10.04	5.57	4.13	23
2	AFT	11.15	9.36	9.19	7.25	4.69	4.81	26
	EFT	8.07	7.44	10.34	7.47	3.52	3.19	29
	PC	9.78	7.38	11.43	10.79	4.09	3.64	23

**Table A4:** Results of repeated measures ANOVAs on the scores of the internalising behaviour, the externalising behaviour, and the social problems syndrome sub-scale of the CBCL, with Time 1 vs. Time 2, and all 3 groups.

Scale	F	*df*	*p*	*η_p_* ^2^
Internalising behaviour	0.22	2.75	0.800	0.006
Externalising behaviour	0.43	2.75	0.650	0.011
Social problems syndrome	1.34	2.75	0.269	0.034

## Appendix D

**Table A5:** Means and standard deviations of the BRIEFs questionnaire’s sub-scales for all groups at Time 1 and Time 2.

Time	Group	Subscales’ Total Raw Scores																*n*
		Inhibit		Shift		Emotional Control		Initiate		Working Memory		Plan/Organise		Organisation of Materials		Monitor		
		*M*	*SD*	*M*	*SD*	*M*	*SD*	*M*	*SD*	*M*	*SD*	*M*	*SD*	*M*	*SD*	*M*	*SD*	
1	AFT	16.54	4.54	15.42	3.81	20.58	5.77	17.31	2.57	23.58	3.11	26.96	4.80	14.73	2.71	17.12	3.65	26
	EFT	18.24	4.84	15.66	3.23	19.59	5.40	17.03	2.96	22.90	3.30	26.97	4.20	13.10	3.22	16.97	2.81	29
	PC	20.09	5.67	16.57	3.84	21.57	5.19	16.39	2.98	23.30	3.05	26.61	4.87	13.61	3.88	18.26	3.41	23
2	AFT	15.88	4.29	14.15	3.80	17.46	5.19	16.31	3.02	22.15	3.34	26.50	4.92	14.12	2.72	15.38	3.49	26
	EFT	16.76	4.45	14.03	2.97	16.52	4.63	15.66	3.36	20.86	3.74	25.76	4.90	12.48	3.33	15.79	3.02	29
	PC	18.78	5.59	14.96	4.12	18.61	5.57	15.96	3.56	21.96	4.38	25.61	5.31	12.96	3.17	16.74	3.91	23

## Appendix E

**Table A6:** Means and standard deviations of the arithmetic task duration and accuracy for all groups at Time 1, Time 2, and Time 3.

Time	Group	Arithmetic’s Task Duration (ms)		Arithmetic’s Accuracy (%)		*n*
		*M*	*SD*	*M*	*SD*	
1	AFT	528	284	84.62	18.86	26
	EFT	569	254	81.61	18.93	28
	PC	505	280	88.23	15.66	24
2	AFT	440	243	88.56	16.99	26
	EFT	554	289	84.38	14.62	28
	PC	419	265	90.10	13.40	24
3	AFT	477	275	86.73	19.54	26
	EFT	516	271	78.57	21.93	28
